# High school training outcome and academic performance of first-year tertiary institution learners - Taking 'Input-Environment-Outcomes model' into account

**DOI:** 10.1016/j.heliyon.2021.e07700

**Published:** 2021-07-30

**Authors:** Bianca Ifeoma Chigbu, Fhulu.H. Nekhwevha

**Affiliations:** Department of Sociology, University of Fort Hare, P.B. X1314, Alice, 5700, South Africa

**Keywords:** High school training, School preparation, Tertiary education, Academic performance, First-year undergraduates, Social-demographics

## Abstract

This study attempted to decipher the link between students' input, output, learning environment, challenges, and students' demographic variables at the tertiary level of education. First-year undergraduate students (n = 122) were surveyed with a structured questionnaire. As hypothesized in the input-environment-outcomes model adopted in this study, students' academic input and learning environment shape learners' study outcomes. Findings revealed that learners' academic performance is influenced by students' demographic variables, intellectual input, educational environment, and challenges, but most importantly, an excellent and effective study environment is what makes the most remarkable difference in the learner's scholastic achievement. If an academic environment does not deliver a broad-quality learning setting to its learners, then it fails in its mission. The educational environment has to gain more influence on how their new learners develop, and the university has to be guarded about how they plan to ascertain that these new entrants become agile learners irrespective of their high school background.

## 1 Introduction

Many high school students frequently find it challenging to adjust to higher education because of inadequate preparation for the tasks ahead in the university. Such responsibilities include increased time slots, assignments, intricate assessment patterns, and more rigorous examinations. Consequently, poor student performance in first-year examinations represents a challenge to the university. There are variables such as students' input, output, environment, challenges, and demographic factors impacting students' successes and failures to universities. Students are unprepared for post-secondary coursework for many reasons, including what was taught in high schools and what is expected in the university (Venezia and Jaeger, 2013). The reason for this could be that, at times, instructors consistently focus on attendance in the classroom instead of evaluating students' participation (Wisneski et al., 2017) and the use of indigenous South African languages in mostly rural high schools as the teaching language (Ajani and Gamede, 2020). Academic success usually relies on several motivational variables that may have a favorable or detrimental effect on academic achievement. The concept of gender or nationality is just part of the academic achievement, likewise instructor's support, school environment, parental interference, and influence from peers (Bal-Taştan et al., 2018).

Mentoring programs received at high school are valuable and a positive part of students' transition to university (Booker and Brevard, 2017). Poynton and Lapan (2017) include emotional and social development as crucial for all successful academic outcomes of the high school students transiting to the university. Thus, positive students' educational outcome is influenced by the school intervention on learners' academic preparation. This intervention is best initiated at a critical transition point in their first few months of school at the university in other to help students gain the necessary skills for academic success (Connolly et al., 2017, p. 4–5). As a result, South African higher institutions are forced to ask questions such as: how does the preparation of students at high school affect students' university adaptation and the factors that impact students’ academic preparation and success in higher education environments?

## 2 Review of literature

### 2.1 High school preparation and tertiary education outcomes

Without guaranteeing that high school graduates are intellectually trained, university enrolment does not place students in a situation where they can excel in higher education (Coca et al., 2017, p. 3) and succeed (Booker and Brevard, 2017). The criteria for identifying underprepared students is their high school low-grade point average (GPA), and these students are considered vulnerable. Again, students with lower self-confidence who struggle to make friends and have a hard time getting along with others may still find it challenging to transition successfully to university education. With such problems, students who possess these high school complications are at greater risk of not reaching the higher institution's expected academic outcome and success (Poynton and Lapan, 2017, p. 375). These scholars further pointed out that many prospective high school student graduates do not plan to pursue post-secondary education. A significant number who are admitted drop out and do not enroll again. Those who register are at a high risk of not being retained or continuing their second university year. Bitzer and Troskie-De Bruin (2004) stated that students' smooth transition from school to higher institutions is still considered a challenge to both segments. For these scholars, the disturbing fact is that students with a lower performance level at school have higher self-confidence than students with excellent school academic outcomes.

It is believed that when students enrol in higher education, they bring personal attributes, social, cultural characteristics, and academic abilities (Tinto, 1993). Additionally, the university enrolment rate could be reduced if a more significant percentage of students graduated at a low educational level and if high schools did not increase their capability (Coca et al., 2017). It is assumed that these young people will tend to aim, register in, and succeed in post-secondary education if their preparation kicks off as soon as possible in high school (Poynton and Lapan, 2017). Researchers and policymakers contend that the main problem is a fundamental misalignment between high school graduation and tertiary academic expectations. Conley (2007, p. 13) noted that being prepared for tertiary education implies having the "information, formal and informal, stated and unstated, necessary for gaining admission to, and navigating within, the post-secondary system." High school graduates with shallow grades are not likely to enroll in any university, which implies that grades are an essential lever to increase access to college (Coca et al., 2017). Hence, the significant predictor of academic success after the first year was high school grades. Strategies that allow first-year students to participate in programs that accelerate social engagement with other students, fast-tracked time management ability, practical learning skills, and different learning strategies can positively impact their academic outcomes. In other words, if first-year university undergraduate students transiting from high school are recognized as early as they arrive and are given structured scholarly attention, their academic performance and GPAs right from their first semester will improve (Connolly et al., 2017). Ignoring these approaches means that high school pupils who are ill-equipped for their tertiary education will be a liability on universities' resources (Falkiner, 2012).

The tertiary system has neglected the selection principles that made it inevitable to achieve high educational excellence earlier. Those who are found to be well qualified for higher education are always allowed to move to the next stage, while unqualified ones were usually left with no choice but to repeat their classes. At present, students who move ahead to university are mostly those whose parents are affluent to bear the burden of such education. Aside from financial influence, learners' input has necessitated students to progress in their academics. Dweck et al. (2011) propose that non-cognitive issues can be an essential element of success at the tertiary level. These issues include keeping an optimistic attitude toward education and persevering when the going becomes rough. Additional problems involve the required obligation to work towards graduation, sponsor the preferred path, find housing, organize transportable to and from the school, and build an appropriate learning environment. This connotes that students can identify learning objectives, formulate plans for attaining them, monitor improvement toward their ambitions, and devise different tactics when the original ones are unsuccessful. According to Mudhovozi (2012, p. 256), new learners need to have their main objectives straightened, re-organize their study methods, and meet academic challenges by focusing and investing quality time in a unique educational setting.

### 2.2 High school graduates' adaptation to the academic environment at tertiary institutions

First-year students from a number of universities in South Africa are typically faced with various difficulties when transferring from high schools to universities. The conditions in the universities are far different from what happens in their high schools. The move from high schools to chosen universities by learners in South Africa is a phase that is full of stressful demands for learners (Ajani and Gamede, 2020). These learners are typically packed with dreams and ambitions to be in universities. Nonetheless, the students indicated that the academic environment they were used to in their high schools was utterly different from their universities (Ajani and Gamede, 2020, p. 15806). First-year learners have to adjust to a changing school environment and a new education that is unfamiliar. The adjustment could be a psychological behavior that helps people to meet environmental demands. New environments tend to generate emotional and physical responses among individuals, and the university atmosphere is part of the challenge because first-year students encounter fear and uncertainty at first.

### 2.3 University acclimatization

Adjustment to the university environment is critical for academic success. Poor academic adaptation is linked to poor academic performance and achievement, which negatively impacts the future success of any student. Academic adaptation in a new study environment is how a student assumes responsibility and thrives on enduring and continuing in any challenging learning situation. However, Petersen et al. (2009) found that student's academic adjustment did not work as an enabling factor of school success and predictor variables. Instead, these scholars indicated that learners psychological factors explained students' academic adjustment than academic achievement. A study at the University of the Western Cape by Moleli (2005) concluded that students' surroundings are not delivering adequate peripheral resources for students to improve vital core resources centering on offering a setting rich in external resources, mainly significant input that will be crucial for an enduring encouraging behavior which encompasses attitudinal change and a high level of academic performance.

The rigorousness of the knowledge and the consequent adjustment differ, giving several features reflecting situational and personal characteristics. Bitzer and Troskie-De Bruin (2004) mentioned that having anticipations of school courses at the entering level is determined by learners' views towards their academic ability based on their high school scores, believing that these outcomes are a suitable display of their future academic success. It is an acknowledged fact that entering the university setting can be terrifying, and this feeling comes with a mixture of responses for most first-year learners. Indeed, first-year students' reaction to this new setting usually influences their academic and social acclimatization at these institutions. Attending lectures always could be an essential aspect of achievement, given that students recognize why they should be there and lecturers doing their very best at what their role entails. For this reason, Poynton and Lapan (2017) argued that teachers responding to students' requests and worries necessitate the students' adjustment to carry on with their studies in their new learning environment.

A structured approach for academic success through students’ first-year experience should be offered in other to support learners through the transition from high school to the institution of higher education – this is to facilitate social engagement with other learners, to promote time management skills, and to practice learning skills and strategies (Connolly et al., 2017). Therefore, it is vital to expand physical facilities, have adequate planning, develop new curricula, increase funding, and provide democratic structures and efficient administration. Also, tertiary education and first-year curricula have to be aligned with those at the high school level as closely as possible. Thus, this underscores the need to develop a diagnostic instrument to assess preparedness for a tertiary institution.

## 3 Input-Environment-Outcomes (I-E-O) model

The study was guided by Astin's Input-Environment-Outcomes (I-E-O) model. For this model, students' inputs (relationship A) are influenced by the students' learning environment (relationship B), and both relationships A and B directly affect students' output/outcome (relationship C). Therefore, the present study investigates the implications of relationships A, B, and C with the aid of different hypotheses, in the research settings, based on Astin (1984) model. The inputs in this study are students' high school preparation; the environment is the academic environment and institutional support available to new students at the university, while the output is the performance in tests and examinations. Learners who enroll in the institution affect the environment, and input can directly affect output or interaction with environmental variables. Therefore, all three variables have effects on each other. According to Astin, the actual learning merit is in the institute's capacity to influence its learners to boost their knowledge and intellectual growth and create a progressive transformation in their lives. In terms of quality evaluation, it is consequently necessary to determine the developments in student bodies over time. For Astin, therefore, aptitude improvement coupled with brilliance can be conditioned by the “quality and quantity of student learning and development.”

## 4 The present study

Scholars have debated the importance of pre-tertiary preparation as an essential factor in determining success or failure in tertiary education. There have also been arguments relating to the relevance of the university environment's challenges as a factor inhibiting students' success and the quality of students' input. The present study attempts to decipher the combined effects of students' pre-tertiary education experiences and their academic input on their university success. The propositions are hypothesized within Astin (1984) model of student development, which past studies have successfully utilized in determining student outcomes (see: Kim and Kutscher, 2021; Patton et al., 2019; Yeung and Fallucca, 2017). Past studies, however, examined only a few variables; thus, fundamental factors such as types of high school, parental income, sources of funding, class, or social status were not explored as the factors contributing to these problems, especially in South Africa and sub-Saharan Africa. Therefore, the present study expands the scope of research into tertiary education preparedness by incorporating the examination of several factors hitherto neglected in studies in sub-Saharan Africa, especially in South Africa.

This study aimed to investigate the effects of high school preparation on the academic performance of first-year undergraduate students. It was, therefore, hypothesized as follows:

i. The experience of the university environment correlates with social demographic factors.
ii. Students' academic input correlates with social demographic factors.
iii. Students' academic output correlates with social demographic factors.
iv. University environmental challenges correlate with social demographic factors.
v. There is a relationship between the university environment and students' academic input.
vi. There is a connection between the academic environment and the scholarly output of students.
vii. The input of the students and the academic output of the students are linked.

## 5 Research method

The first-year undergraduate students were sampled. The total number of first-year students was 973. Using the *Raosoft* sample size calculator (@ http://www.raosoft.com/samplesize.html) with a 5 % error margin, 95 % confidence interval, and 10 % response distribution, a sample size of 122 respondents was derived. The sample sizes were based on the proportion of total students in each faculty. 34.4 percent of responses were from the faculty of Science and Agriculture, 31.3 percent from the faculty of Social Science and Humanities, and 18.8 percent from Management and Commerce and Education faculties, respectively. This is because the faculty of science and agriculture and social sciences and humanities have the most students enrolled in the university. In contrast, the faculties of education, management, and commerce have a low number of registered first-year students.

Data was collected using a self-administered structured questionnaire with Likert scale question styles. Data were analyzed using IBM SPSS. Besides the social-demographic factors, more than one questionnaire component was used to evaluate certain variables in this study. Consequently, as the first step in data analysis, the Principal Components Analysis (PCA) was adopted to reduce multiple questionnaire items into scales. The following four levels were constructed:

i. Environmental Challenges (ENVCHAL)
ii. Students' Input (STUDINP)
iii. Student Output (STUDOUT)
iv. University Environment (UNENV)

The results of PCA for all four variables are presented in the result section, showing descriptive statistics, Kaiser Meyer Olkin (KMO) test of sample size adequacy, and Bartlett's Test of Sphericity (BTS). Cronbach alpha scores indicating scale reliability are also reported. This study was approved by the University of Fort Hare's Research Ethics Committee.

## 6 Results and findings

Four different subscales were constructed in line with Astin (1984) model to determine the factor contributing to first-year student performance and output. The results derived from factor analysis are presented below.

### 6.1 Environmental challenges (ENVCHAL)

The ENVCHAL subscale was constructed using 11 questionnaire items relating to the university environment challenges. Responses (n = 122) were measured on a Likert scale of 1–5, where 1 = Strongly Disagree and 5 = Strongly Agree. The highest possible score was 55, and the lowest was 11. Higher scores indicate a higher perception that the university challenges are different from those of high school.

A principal Component Analysis (PCA) was computed, and one factor, environment challenges (ENVCHAL), was extracted electronically, with a KMO of 0.520, BTS, X^2^ = 67.080 (df = 55), p < 0.05 (see Table 1) indicating that the sample was adequate for factor reduction. Scale reliability statistics show a ‘good’ Cronbach's alpha of 0.80, with scale mean = 38.36, SD = 1.99. ENVCHAL was saved and utilized in further bivariate analyses. As Table 1 shows, ENVCHAL accounts for 59.617 % of the variance in the factor. Other possible factors become relatively insignificant after ENVCHAL was extracted, therefore lessening the need to generate further sub-dimensions.

**Table 1:** KMO, BTS, Extracted Variance and Cronbach Alpha scores for all Scales.

	KMO	BTS	DF	Cronbach's Alpha	Total Variance Explained (%)	Sig.
ENVCHAL	.520	67.080	55	0.80	59.617	.000
STUDINP	.512	393.759	55	0.68	76.517	.000
STUDOUT	.505	387.347	36	0.66	79.714	.000
UNENV	.739	1110.200	66	0.80	82.077	.000

### 6.2 Students’ input (STUDINP)

The STUDINP subscale was created using 11 questionnaire items relating to the students' input. Responses (n = 122) were measured on a Likert scale of 1–5, where 1 = Strongly Disagree and 5 = Strongly Agree. The highest possible score was 55, and the lowest was 11. Higher scores indicate higher students' perception of their academic input. Principal Component Analysis (PCA) was computed, and one factor, Students' input (STUDINP), was extracted electronically, with a KMO of .512. BTS, X^2^ = 393.759 (df = 55), p < 0.05 (see Table 1) indicating that the sample was adequate for factor reduction. Scale reliability statistics show a ‘good’ Cronbach's alpha of 0.68, with scale mean = 41.8.93, SD = 3.16. STUDINP was subsequently saved and utilized in further bivariate analyses and tests of the hypotheses. As Table 1 shows, STUDINP accounts for 76.517 % of the variance in the factor. Other potential elements become fairly insignificant after STUDINP was obtained, thus diminishing the need to create additional sub-dimensions.

### 6.3 Students’ output (STUDOUT)

The variable STUDOUT was designed to conceptualize the outcome of students' engagement in campus activities. Outcomes usually include test results and processes that lead to graduation. The STUDOUT subscale was constructed using nine questionnaire items relating to the students' output. Responses (n = 122) were measured on a Likert scale of 1–5, where 1 = Strongly Disagree and 5 = Strongly Agree. The highest possible score was 45, and the lowest was 9. Higher scores indicate higher perception of the students' output in their studies. Principal Component Analysis (PCA) was computed, and one factor, Student Output (STUDOUT), was extracted electronically, with a KMO of .505, BTS, X^2^ = 387.347 (df = 36), p < 0.05 (See Table 1), indicating that the sample was adequate for factor reduction. Scale reliability statistics show a ‘good’ Cronbach's alpha of 0.66, with scale mean = 34.70, SD = 1.65. STUDOUT was subsequently saved and utilized in further bivariate analyses and tests of the hypotheses. As Table 1 shows, STUDOUT accounts for 79.714 % of the variance in the factor.

### 6.4 University environment (UNENV)

The variable UNENV centers on the tertiary education environment. UNENV deciphers the effects of the inadequacies in tertiary institutions' preparedness to support learners' quest for academic success adequately. The UNENV subscale was formed using 12 questionnaire items relating to the students' environment. Responses (n = 122) were measured on a Likert scale of 1–5, where 1 = Strongly Disagree and 5 = Strongly Agree. The highest possible score was 60, and the lowest was 12. Higher scores point to a higher perception of the students' environmental challenges in their studies. Principal Component Analysis (PCA) was computed, and one factor, Environment (UNENV), was extracted electronically, with a KMO of .739, BTS, X^2^ = 1110.200 (df = 66), p < 0.05 (See Table 1), demonstrating that the sample was sufficient for factor reduction. Scale reliability statistics show a ‘good’ Cronbach's alpha of 0.82, with scale mean = 42.39, SD = 3.20. UNENV was subsequently saved and utilized in further bivariate analyses.

### 6.5 ENVCHAL, STUDINP, STUDOUT, and UNENV correlate with socio-demographic factors of students

Different variables are determining the level of integration and adaptation of new students to the tertiary environment. Thus, the variable environmental challenges (ENVCHAL), students' input (STUDINP), students' output (STUDOUT), and university environment (UNENV) are analyzed according to the respondents’ demographic characteristics. These four variables were saved in the SPSS program after PCA. To determine how the four factors correlated with socio-demographic variables of students, bivariate correlations were conducted to determine how respondents differed by socio-demographic.

To define the demographic factors in Table 2 above, in the context of education, gender applies to the socially defined attributes of femininity and masculinity of learners and the expectations placed on both female and male students' academic output. A student's age is the age at which he or she registered at the learning institution, as determined by the date of birth on their academic record. The place of origin of a student represents the birthplace of the student. Parents' income is the amount of every form of payment made to a parent and used by the parent to cover the family's usual living expenses, including college expenses. A student's financial sufficiency is when the learner shows that they have ample financial capital to pay tuition fees, commuting costs, and living expenses for the length of their studies. School type indicates private or public school, while school size considers the number of students in the school. Place of high school includes if the school is situated in a rural or urban area. Academic grade, also known as grade point average, is a statistic that represents the average amount of a student's cumulative final grades achieved in classes over time. It is determined by adding all accrued final grades and dividing the sum by the number of grades given to students to complete their schooling.

**Table 2:** Zero-order correlations for ENVCHAL, STUDINP, STUDOUT and UNENV and demographics.

	1	2	3	4	5	6	7	8	9	10	
(1) Gender											
(2) Age											
(3) Place of origin											
(4) Parents income											
(5) Financial Sufficiency											
(6) School type											
(7) Place of high school											
(8) Language											
(9) Student's grade point average											
(10) School size											
(11) ENVCHAL	479∗∗	-.236∗∗	.300∗∗	-.476∗∗	.568∗∗	.575∗∗	-.578∗∗	.278∗∗	.575∗∗	.578∗∗	-
(12) STUDINP	-.436∗∗	-.308∗∗	-.183∗	.532∗∗	-.378∗∗	-.480∗∗	.426∗∗	-.146	.579∗∗	.218	-
(13) STUDOUT	-.143	-.491∗∗	-.291∗∗	-.065	-.076∗∗	.192	-.236∗	.191∗	.103	.218∗	-
14) UNENV	.210∗	.795∗∗	-.136	.430∗∗	-.289∗∗	-.397∗∗	-.196	.027	.131∗	205∗	-

∗∗. p < 0.01 (2-tailed), ∗. p < 0.05 (2-tailed).

Results of zero-order correlations (see Table 2) showed that students' origin, gender, parent's monthly income, financial sufficiency, high school type, place of high school, school grade, the language taught, high school size, and age has a significant relationship with university environmental challenges. Therefore, university environmental challenges correlate with the socio-demographic factors of the students.

Further results, as shown in Table 2, indicate that STUDINP is correlated with gender, age, parent income, financial sufficiency, type of high school, and school grade. However, the relationship between STUDINP and place of school and place of origin are insignificant.

More findings in Table 2 revealed that STUDOUT is associated with age, place of origin, financial sufficiency, and language taught. However, the relationships between STUDOUT and high school place and size of school are insignificant. Again, it was revealed that UNENV is correlated with age, parent income, finance sufficiency, and school type. Though, the relationships between UNENV and gender, school grade, and size of school are insignificant.

### 6.6 The input-output-environment determinants of students’ performance

It has been proposed that high rates of resilience are required for successful university transition. In South Africa, Nel et al. (2009) found that parents' lack of educational support and goals was a substantial dissuasion for first-generation students. The analysis culminated in determining the correlations between university environment, challenges, input, and output.

The students' socio-demographic factors were obtained for the relationships between UNENV, STUDINP, ENVCHAL, and STUDOUT. The results of the association are depicted in Table 3. The bivariate correlation results showed that UNENV is positively correlated to STUDOUT. This result implies that the university environment does predict the quality of the academic output of the students. In other words, environmental influences determine whether students obtained desired educational outcomes and the time it took to obtain the desired result. Other bivariate correlations further showed that UNENV is correlated to STUDINP.

**Table 3:** Zero-order correlations for environment, input, output, and challenges.

		Student Output	University Environment	Challenges	Students Input
Student Output	Pearson Correlation Sig. (2-tailed)	1	-.501∗∗ .000	.040 .664	.219∗ .004
University Environment	Pearson Correlation Sig. (2-tailed)	-.501∗∗ .000	1	-.177 .052	-.230∗ .004
Challenges	Pearson Correlation Sig. (2-tailed)	.040 .664	-.177 .052	1	-.418∗∗ .000
Students Input	Pearson Correlation Sig. (2-tailed)	.219∗ .004	-.230∗ .004	-.418∗∗ .000	1

∗∗. Correlation is significant at the 0.01 level (2-tailed).

∗. Correlation is significant at the 0.05 level (2-tailed).

Bivariate correlations also examined the relationship between students' input (STUDINP) and students' academic output (STUDOUT). The result in Table 3 showed that there is a minimal relationship between the academic input and academic output. Consequently, there is a relationship between the study environment and the students’ academic input.

With a weak correlation coefficient of 0.219 between students' academic input and academic output, surprisingly, this indicates that academic input is not that important for predicting the academic outcome of students – STUDOUT could be better correlated with the combination of UNENV, STUDINP, and ENVCHAL. Nonetheless, it is confirmed that a relationship exists between STUDINP and STUDOUT regardless of its strength. The bivariate correlation findings further uncovered that ENVCHAL did not associate with STUDOUT, but it is strongly related to STUDINP. Based on these results, the university environment of learners, the challenges encountered by the learners, the amount of energy, effort, dedication, and encouragement students commit to their educational activities define their personal academic development and understanding. Also, profound learning experiences provided through lectures promote student engagement which has an important connection with students desired university output.

In Table 4 below, these students indicated that among the challenges they are experiencing are the long hours of study in their new environment. University hours of study can be overwhelming for learners in a completely new environment. Adjustment and acclimatization to get over the initial long-hour culture shock are usually challenging to embrace for new learners, which they believe influence their learning outcome. Students who are prepared to face the challenges of attending a higher percentage of their classes, lectures and spend more time studying are most likely to achieve better academic outcomes. Again, this study indicates that students often attach the rate of social life at the university to their academic success. They believe that when the university environment lacks good social life, this tends to hamper the input and outcome of its students. Factors constituting ‘university environment’ may be a combination of social and physical aspects that directly and indirectly affect students' performance. However, many students struggle not because they socialize less, but some students are distracted with higher socialization expectations and unable to comprehend that more academic responsibilities take the lead against social activities in tertiary settings.

**Table 4:** Descriptive statistics for ENVCHAL, STUDINP, STUDOUT, and UNENV.

Variables	Survey questions	Mean	Std. deviation	Components
**ENVCHAL:** Highest score Lowest score	Study hours in university are too long compared to high school I go to class without reading my previous notes	4.35 3.22	.615 .724	.499 .576
**UNENV:** Highest score Lowest score	Social life at the university is inadequate There is a language barrier	4.39 1.93	.745 1.002	.805 .690
**STUDINP:** Highest score Lowest score	I really like being a university student I am actively involved in university extracurricular activities	4.55 2.79	.500 .911	.653 .858
**STUDOUT:** Highest score Lowest score	I am going to get a good result at the end of this semester I have my own timetable aside from the lecture timetable	4.32 3.08	.549 1.001	.779 .856

The students indicate further that the joy of being among university learners and their physical/emotional effort and involvement drive their academic input. This result shows that most students have a positive perception of their opportunity to be university students. As a result, their motivation to succeed in higher education is improved. Students who have positive academic self-perceptions tend to employ more effort in school in general and their specific academic subjects and responsibilities, which can facilitate learning and performance outcomes. Students believe that they have made an effort and nurtured the ability to do well in academics. These learners have confidence that they already possess enough brainpower to gain good results and be intrinsically motivated to challenge themselves for better academic success.

## 7 Discussion

An excellent academic performance, especially from those learners transiting from high school to the first-year undergraduate in higher education institutions, has been reduced to various demographic factors. In this study, the demographic variables of interest can be summarized to include parental income, students' origin, gender, financial sufficiency, high school type, place of high school, school grade, the language taught, high school size, and age. Academic environmental challenges commonly experienced by students in their first year of university pose significant obstacles to socialize, adapt, and study. Challenging learning issues comprised learning difficulties, balancing study time, underachievement, lack of assimilation, bullying, presentation/exam/test anxiety, overwhelmedness, homesickness, lack of sense of self-worth (fear of expression and sense of style), and depression. Hypothesizing within the I–O-E model, the present study's result confirmed that first-year students' demographic factors correlated with environmental learning challenges. Students relying upon the self-study method, campus-provided tutors, and peers have been strategies for overcoming environmental learning challenges (Wisneski et al., 2017).

The study hypothesized that students' input correlates with their social demographic factors. Student's inputs are those unique qualities that accompany students to the university during the entry period (Astin, 1993, p. 18), including their attitude, resilience, and motivation to learn in their first year of study in the university, which was developed in their high schools. The indication is that students' study habits formed in high school persist in their university life. These results concur with Geiser and Santelices (2007) that learners who previously performed very well in high school also performed much better in tertiary institutions and that parents' role stimulates academic performance. Students' optimistic attitudes as intellectual input into courses and university choice are promising and reflect the value of higher achievement (Terzi and Kirilmazkaya, 2020).

Moreover, these optimistic behaviors may be accomplished by enhancing the standard of pre-university education. A positive attitude can come in students' higher resilience and learning style, influencing academic achievement (Shirazi and Heidari, 2019; Van Hoek et al., 2019). Students' resilience as an input factor also predicts intention to leave school, academic success, and dropout (Van Hoek et al., 2019). Socioeconomic status has been an essential predictor of the input of the students to access post-secondary institutions. Coca et al. (2017) added that higher socioeconomic status is associated with increased exposure to college financial resources and broader access to social networking and resources that support student college preparation. Mudhovozi (2012) discovered that financial difficulties mainly affect male learners than female learners. Generally, it can be argued that a higher percentage of learners solely depend on student loans to acquire a degree. Hence, a significant number of students cannot see themselves through tertiary education if the student loan is to be removed. This is because many South African university learners battle financial difficulties while living in abject poverty (Lloyd and Turale, 2001).

In our study, high school's location and size did not predict academic outcomes in the university. This means that learners' educational outcome does not depend on how big the school is or its location. However, other demographic variables of students were identified to relate to their academic output. Contrary to our findings, Considine and Zappalà (2002) and Thiele et al. (2016) indicated that learners from rural areas and remote backgrounds are more likely to have a lower academic performance to drop out than students coming from urban areas. The reason is that these students face academic difficulties such as higher education academic writing, teaching and studying in English, and lack of computer skills (Ajani and Gamede, 2020) - such schools are commonly found in African areas (Wickham et al., 2008).

Correlating with the results of this study indicating that the first-year academic outcome depends on the language used in teaching in prior school, Alsahafi and Shin (2017) reported that language abilities are the biggest obstacle to the academic and social transition specific students. The reason for this barrier is that the medium of instructions at their rural high schools had been the use of indigenous languages, where their lessons had been taught in IsiZulu, IsiXhosa, Sesotho in most South African rural high schools - thus, incapacitated their learning (Ajani and Gamede, 2020, p. 15810). Teaching in these indigenous languages resulted in learners’ fears of using English as a medium of expression, making it difficult for them to comprehend and express their views through academic writing (Ajani and Gamede, 2020). These students can cope by improving their language competence (Alsahafi and Shin, 2017).

In this study, gender influences the learning outcome of students. Gender is a predictor widely used for educational outcomes; however, scholars such as Bal-Taştan et al. (2018) and Terzi and Kirilmazkaya (2020) revealed that gender has little impact on predicting academic performance. Nonetheless, other studies have linked academic performance and GPA to students’ gender (Ferrão and Almeida, 2019; Lemmens et al., 2011). To differentiate between male and female academic performance, Adekitan and Shobayo (2020) and Thiele et al. (2016) explained that female students prefer to boost their academic success from the first to the last year of school than their male peers. From the first to the final year, the female students' performance improved significantly in the first-class group. However, their numbers in the second-class upper group remained relatively constant in the last year compared with their first-year performance. More male students slipped from the second class upper to the second class lower group, demonstrating a decline in academic performance (Adekitan and Shobayo, 2020). This indicates that significant changes occurred between the period of admission and the first year in both male and female students' performance up to the final year. These differences in academic achievement may be due to lack of concentration as a result of factors such as distractions on the Internet, excessive playing of computer games, peer pressure, and other social activities of male students, which provide significant avenues of distraction from the academic demands of a university (Adekitan and Shobayo, 2020). Again, these differences in educational achievement between males and females may be related to Poynton and Lapan (2017) study, which reported that female learners receive more support from school counselors than male students in preparing for their future careers.

Just like the present study, the university entrance score and high school education are among the predictors of academic performance (Ferrão and Almeida, 2019; Terzi and Kirilmazkaya, 2020). Although the association between GPA and university entrance scores ranges spontaneously through courses (Ferrão and Almeida, 2019) - some courses have more vital prerequisites for previous preparation than others, with potential curriculum development (Thiele et al., 2016). Students' futures at university are also uniquely reliant on school credentials, but these themselves are constrained as predictors of academic performance (Thiele et al., 2016). Learners who attended schools deemed high-performing in terms of A-level performance joined higher A-level colleges relative to those who attended lower-level schools. Even though accelerating learning skills and helping students fresh from high school can increase their GPAs in their first semester at the university, it is unlikely that this strategy will prevent some students from getting low grades for the next semester (Connolly et al., 2017).

Socioeconomic level, student's nationality, and place of origin were identified in this study as some of the variables that impact learners' academic success. Regarding the place of birth, Bal-Taştan et al. (2018) identified it to influence the level of students' academic achievement. Ferrão and Almeida (2019) concurs with the socioeconomic aspect of our result. They reported that students whose parents have little more than a lower secondary education had enhanced incentive to reflect students' willpower from socio-economically deprived classes who have been accepted to the university and seek to obtain successful outcomes since good performance is a prerequisite to retain a scholarship. Better still, there is a rise in academic performance, as the educational standard of both parents rises from a lower level to a higher stage (Terzi and Kirilmazkaya, 2020). However, a higher level of education on the mother's part enhances students' achievement rather than a higher education level on the father's part. In general, students from higher socioeconomic classes have a consistent superiority over low socioeconomic class students in academic results (Destin et al., 2019). The overwhelming majority of the current socioeconomic divide is expected to be shaped by the underlying causes of inequalities (Destin et al., 2019).

The present study results showed that the university's environment is positively correlated to students' input. This implies that the better the university environment, the more effort the students will put into their academic work. Educational backgrounds are responsible for preparing young learners intellectually and adjusting them (input) to the university's new demands. These results are similar to a study by Considine and Zappalà (2002) that the university environment influences students' learning styles. Students reported that course interactivity and format of the class as criteria were critical motivations for enrolling in specific courses (Wisneski et al., 2017). Scholars, for example, Tinto (1993), argued that tertiary education administrators must understand students' academic abilities, personal attributes, and socio-cultural characteristics on their arrival and enrolment at the institutions. Dweck et al. (2011) concluded that dedication, motivational or non-cognitive factors are related to success at the tertiary level. Social and physical support programs give value to the social and physical inspiration of the students. Loots (2009, p. 211) showed that support from friends, academic encouragement, and a combination of in and out of class interaction and extracurricular activities enhance school performance. Working with mentors has allowed students insight into essential academic and life skills such as study strategies, time management, and promoting the growth and maturation of young learners by providing emotional assistance and encouragement (Booker and Brevard, 2017). This is why Strydom et al. (2010) concluded that when students interrelate with academics inside and outside the classroom, they absorb how their teachers make decisions and their ways of resolving problems.

The findings based on this study also showed that the university's environment is correlated to students' output. The present result agrees with Loots (2009) study that friendly support, administration of the institution, safe environment, the attitude of lecturers improved students' self-reliance to achieve a better outcome. The value of education is determined by the quality of the education system to which a student is exposed (Adekitan and Shobayo, 2020). In reality, the influence of instructor characteristics at the level of self-efficacy is critical for education and student achievement (Bal-Taştan et al., 2018). Integration with the university environment and a greater focus on classes over college years will help students successfully raise their GPAs and boost their educational attainment (Shirazi and Heidari, 2019). To ensure continuous relevance as an institution and to produce graduates that can meaningfully impact society after graduation, a higher education institution must develop quality assurance systems that track institutional efficiencies and practices to identify operational gaps toward proffering improvement (Adekitan and Shobayo, 2020).

The assumption made by Astin's model that the outcome of student development was enriched by attending a high-quality institution (environment), therefore, confirms the findings of the present study. Conley (2007) noted that the learning environment's satisfactory conditions make it possible for students to accomplish their educational goals and targets. Regardless of the findings of this study, the universities need to lend a helping hand to new students in terms of their adaptation to the new environment they are about to face. As Astin (1984) indicated, the best institutions add value to students' experience, abilities, manners, and individual maturity. Prebble et al. (2004, p. 65) proposed that university administrators should prioritize providing services that will adapt, integrate, and equally support new students to enhance excellent learners' academic outcomes.

The results of this study also revealed that student academic inputs are positively correlated to student intellectual output. Hence, there is an essential connection between students' attitude, resilience, motivation to learn, study habits, study engagement, and student desired university output. This result is supported by Lemmens et al. (2011, p. 615) argument that students' motivation, the educational dedication of learners, and the ability to learn are directly linked to academic performance. As reported by Ajani and Gamede (2020), the input of students, which includes students' backgrounds of lack of computer knowledge and skills; low high school workload; changes in their social lives different from their high school days; use of indigenous languages for teaching in high schools as against the use of English now at the university was responsible for their hindrances to adjust to transitional changes at the university. Universities should run programs with good course content and balanced non-academic social aspects (Adekitan and Shobayo, 2020) – this is to steer them away from potential failure and put structures in place to help students achieve their target grades.

In summation from our statistical analysis, students' academic performance is influenced by students' demographic variables, intellectual input, educational environment, and challenges. Most importantly, a good and effective study environment makes the most significant difference in the learner's scholastic achievement. If an academic environment does not deliver a broad-quality learning setting to its learners, it fails its mission. Each study environment that students choose is expected to influence their ability to focus, remember, concentrate, and persist through learning difficulties. For instance, the continuous teacher learning process, mastering new pedagogies and teaching skills, will help students achieve a higher learning level. The educational environment has to gain more influence on how their learners develop, and the university has to be guarded about how they plan to ascertain that their students become agile learners.

## 8 Conclusion

This study shows that the time students invest in their academic activities and involvement can be measured using qualitative and quantitative measures. This indicates that the quality of the students' input, which is associated with their academic and family background, hugely impacts their academic outcomes negatively or positively. Those students whose families are in difficult financial straits or are not being sponsored by scholarships or bursary to aid students in covering the cost of tertiary studies would struggle with insufficient finances to see them through tertiary education. The opportunity of being accepted in a university improves students' motivation to succeed in higher education and in their specific academic subjects. Students often attach the rate of social life at the university to their academic success. However, many students high school graduates transiting to higher education institutions struggle not because they socialize less. Still, some students are distracted with higher socialization expectations and unable to comprehend that more academic responsibilities take the lead against social activities in tertiary settings. New students encounter pressure, stress, uncertainties, difficulties, and unsure preparation in higher educational settings. Although these new learners struggle to cope and overcome or manage these challenges, some find the struggle and the challenges impossible to bear and are overwhelmed by the rigid experience. Combining students' academic input, output, and the environment variables makes it possible to predict students’ performance in their learning environment instead of looking at only one variable.

## 9 Recommendations

The challenges that first-year students face have the potential of negatively affecting their educational outcomes. It is, therefore, imperative for tertiary institutions to strategically manage new students. Accordingly, the study suggests that high schools, university authorities, and students should devise a preparation strategy around the performance variables to make the first-year students more prepared and adjusted to perform excellently in higher learning institutions. One strategy is for the learning environment (high school teachers, lecturers, and instructors) to concentrate on rigor and encouragement for students by motivating them not to focus on the materials provided at school but to expand their academic horizons through personal learning and readings. Nevertheless, the instructors as an expert will attempt to reveal to their learners how to maneuverer such learning techniques. University authorities can support students to prepare to produce outstanding academic results. This can be done by recommending and giving students the ability to share phone numbers and emails – helping learners build, for example, SKYPE personal identity number, WhatsApp chat community, ZOOM group, and collaborative academic climate discussions. Learning strategies such as these are flexible and can happen on and off the university environment.

Again, the study environment should teach curricula and train its learners on various methods to consume information and optimize the benefits of learning time for both fast and sluggish learners. Thus, softly coaxing the learning route to create novel study techniques and mixing them with personal study methods or selecting the most successful study technique. In addition to the suggestions, throughout the lecture time, it is believed that all students, regardless of their degree of comprehension, can better grasp the course if the lecturer uses fewer academic jargon and uses more of the most exact possible words. Thus, explaining complicated technical issues and concepts utilizing vocabularies and phrases can be interpreted by ordinary learners. University lecturers may make extra efforts in preparing audio or video recordings for slow learners, which they can play and listen to at their convenience. Finally, it is suggested that future studies should focus on fewer influencing factors of learners’ academic output – more from a psychological perspective and with the implementation of open-ended qualitative questions, which have not been dealt with in this study and thus; limited our study.

## Declarations

### Author contribution statement

Bianca Ifeoma Chigbu, Fhulu H Nekhwevha: Conceived and designed the experiments; Performed the experiments; Analyzed and interpreted the data; Contributed reagents, materials, analysis tools or data; Wrote the paper.

### Funding statement

This research did not receive any specific grant from funding agencies in the public, commercial, or not-for-profit sectors.

### Data availability statement

The authors do not have permission to share data.

### Declaration of interests statement

The authors declare no conflict of interest.

### Additional information

No additional information is available for this paper.
